# Suspended Fœtal Animation

**Published:** 1872-02

**Authors:** A. W. Griggs

**Affiliations:** West-Point, Ga.


### SUSPENDED FŒTAL ANIMATION.

BY A. W. GRIGGS, M.D., WEST-POINT, GA.

The causes which operate in the production of asphyxia in newly-born infants are of prime importance, since, in some cases, they may be either avoided, set aside, or so modified as to result only in partial effects; yet it is clearly evident that many instances occur in which the most philosophic and the best-directed efforts of the accoucheur will prove unavailing. Compression of the fœtal brain, of the placenta, of the prolapsed funis, hemorrhage from placental detachment, too great an interval between the expulsion of the head and that of the trunk, the administration of secale cornutum during labor, and the free and injudicious exhibitions of anæsthetic vapors (especially of chloroform), are reckoned by authors as the principal causes, direct or indirect, which produce or conspire to the production of suspended fœtal animation. There may be obliquity or contraction of the pelvis; the latter may be preternaturally small without irregularity in its diameters, as with dwarfs. There may be resistance by tumors or unyielding perineal muscles, or by organic vaginal obstructions from adventitious formations within, or cicatricial contractions of this canal from previous ulcerations. The fœtal head may be unnaturally large, with or without corresponding bulk of the trunk, or there may be malposition or malpresentation. You will readily perceive that two or more of these conditions, if present, would jeopardize the life of the mother, as well as that of the child.

As it would be foreign to the purpose of this paper to enter into a minute account of cases in which the fœtus cannot be expelled per vias naturales, without manual or instrumental assistance, I shall content myself by merely alluding to the fact that the Cæsarean operation, if performed in time, would save children whose heads can only partially pass within the brim, there to suffer the increasing certainty of dangerous compression from the active throes of labor, and also that the timely and skillful application of the forceps would save many whose heads pass through the brim, and are impacted in the inferior strait; for it is worthy of remark, that the uterine contractions are, to a great degree, proportionate to the obstructions opposing delivery. Inordinate and resistless impressions are reflected, arousing every expulsive energy, until the overwrought powers and forces of the system induce inertia uteri, with its fearful symptoms; or rupture of the uterine parieties or perineum may occur, and also fœtal life be destroyed. The laws to be observed and the practice to be instituted in such cases are clearly set forth by most obstetric writers, and I only insist on their attentive observance and prompt execution. The laws also regulating the conduct of *labors* with placenta prævia, partial or complete, or of those where there are malpositions or malpresentations, are sufficiently elaborated by writers on this subject; and I would not refer to them if I were not satisfied that the life of the fœtus is often sacrificed by the tardiness of the accoucheur, in the exercise of his high prerogative.

Suffer me now to introduce that class of cases wherein compression is produced by muscular rigidity—which, I think, is the most common difficulty with which we have to contend in obstetric practice.

Tartar emetic in nauseating doses, frequently repeated, is, in my judgment, the safest and most efficient remedy that can be employed; auxiliary to this, the constant application of cloths, wrung out of warm water, to the vulva and perineum, will be highly beneficial. In this there is nothing new or original; the treatment supplies the indications, and has been crowned by universal success in my practice for a good many years.

Asphyxia is caused by protracted compression of the thoracic viscera, when too much time elapses between the expulsion of the head and that of the trunk. My rule, in such cases, is to wait thirty or forty seconds after the birth of the head, and if no pain begin, dip my left hand in cold water and apply it over the uterus;—use gentle friction; and if the contraction is still wanting, or insufficient, I immediately introduce the right index finger, pass it to the fœtal axilla, and lift the shoulder over the fourchette, supporting the weight of the head upon the palm and wrist of the right hand. The nature and seat of vaginal obstructions will suggest to the practitioner the indications to be met, and no valuable time should be lost in attempting their modification or removal; for delay is more dangerous to fœtal than maternal life in such cases. I have no doubt but that secale cornutum is fully capable of producing asphyxia in the fœtus, when unscientifically given. The well-known power of this drug in constringing the coats of the blood-vessels, superadded to the increased tonicity of uterine contractions, under its use, presents a combination potent for evil to mother and offspring—diminished supply of blood and powerful compression of vital organs. Nevertheless, it is, when indicated, also potent for good, preventing the dangers of delay which sometimes arise. Anæsthetics superinduce fœtal asphyxia by obstructing the oxygenation of the mother's blood, and are mostly unsafe, and should be discarded altogether, or prescribed with wise discrimination and administered with prudential care.

(TO BE CONTINUED IN ANOTHER ARTICLE.)

